# Activin a promotes myofibroblast differentiation of endometrial mesenchymal stem cells via STAT3-dependent Smad/CTGF pathway

**DOI:** 10.1186/s12964-019-0361-3

**Published:** 2019-05-17

**Authors:** Zhenzhen Zhang, Jing Wang, Yabing Chen, Luxuan Suo, Huixian Chen, Li Zhu, Guiping Wan, Xiaodong Han

**Affiliations:** 1 Immunology and Reproduction Biology Laboratory & State Key Laboratory of Analytical Chemistry for Life Science, Medical School, Nanjing University, Nanjing, 210093 China; 2 Jiangsu Key Laboratory of Molecular Medicine, Nanjing University, Nanjing, 210093 China; 3 Affiliated Hospital of Integrated Traditional Chinese and Western Medicine, Nanjing University of Chinese Medicine, Nanjing, 210028 China; 4 Jiangsu Province Academy of Traditional Chinese Medicine, Nanjing, 210028 China

**Keywords:** Endometriosis, Activin a, Fibrosis, SUSD2, Peritoneal fluid

## Abstract

**Background:**

Endometriosis, characterized by the presence of functional endometrial tissues outside the uterus, is one of the most common gynecological disorders. Endometrial mesenchymal stem cells (MSCs) are crucial for the occurrence and development of endometriosis. Ectopic endometrial MSCs exist in the peritoneal cavity. Thus, the bioactive factors in endometriotic peritoneal fluid may regulate the biological behaviors of endometrial MSCs.

**Methods:**

In this study, after assessing the concentration of Activin A in peritoneal fluid using ELISA, we isolated and cultured endometrial MSCs and investigated whether Activin A stimulated endometrial MSCs to differentiate into myofibroblasts and clarified the underlying mechanisms by quantitative real-time PCR, Western blot analysis, immunofluorescent staining, RNA interference and Chromatin immunoprecipitation. We also employed the inhibitors of Activin A to explore the possibility of suppressing the development of fibrosis in endometriosis using primary endometrial MSCs cultures and a mouse model of endometriosis.

**Results:**

Here, we revealed that Activin A significantly elevated in endometriotic peritoneal fluid and activin receptor-like kinase (ALK4), the specific receptor for Activin A, obviously enhanced in ectopic endometrial MSCs compared with eutopic endometrial MSCs from women with or without endometriosis. Next, we found that Activin A drived myofibroblast differentiation of endometrial MSCs, with extremely enhanced expression of connective tissue growth factor (CTGF). CTGF was shown to be required for Activin A-induced expression of *ACTA2*, *COL1A1* and *FN1* in endometrial MSCs. CTGF induction by Activin A in endometrial MSCs involved the activation of Smad2/3, as evidenced by the phosphorylation and nuclear translocation of Smad2/3 as well as the binding of Smad2/3 to CTGF promoter. Furthermore, Smad/CTGF pathway in endometrial MSCs required activation of STAT3 while independent of PI3K, JNK and p-38 pathways. In addition, we also demonstrated that inhibition of Activin A pathway impeded myofibroblast differentiation of endometrial MSCs and ameliorated fibrosis in endometriosis mice.

**Conclusions:**

Activin A promotes myofibroblast differentiation of endometrial mesenchymal stem cells via STAT3-dependent Smad/CTGF pathway. The results provided the first evidence that STAT3 acted as a crucial Activin A downstream mediator to regulate CTGF production. Our data may supplement the stem cell theory of endometriosis and provide the experimental basis to treat endometriosis-associated fibrosis by manipulating Activin A signaling.

**Electronic supplementary material:**

The online version of this article (10.1186/s12964-019-0361-3) contains supplementary material, which is available to authorized users.

## Background

Endometriosis, histologically characterized by dense fibrosis in and surrounding the endometriotic lesions, is one of the most common gynecological disorders affecting 10–15% of all women of reproductive age [1]. Although the definitive mechanism in the etiology of endometriosis remains uncertain, considerable evidences indicate that endometrial stem cells, which have been identified in menstrual blood and as clonogenic cells in ectopic lesions, are crucial for the occurrence and development of endometriosis [2]. Endometrial mesenchymal stem cells (MSCs), which can be isolated as Sushi Domain containing-2 (SUSD2) positive cells, reconstitute endometrial stroma when xenografted under the kidney capsule of mice, indicating their regenerative potential [3]. Endometrial MSCs derived from endometriosis patients express increased extracellular matrix proteolysis genes in comparation with normal women [3]. Ectopic endometrial MSCs exhibit higher proliferation potential than matched eutopic samples [4].

We recently found that ectopic endometrial MSCs expressed elevated fibrotic proteins, including collagen I, α-smooth muscle actin (α-SMA), fibronectin, and connective tissue growth factor (CTGF), compared with eutopic endometrial MSCs from women with or without endometriosis and that endometriotic peritoneal fluid promotes myofibroblast differentiation of endometrial MSCs (In Press). Myofibroblasts are the main source of extracellular matrix in fibrosis. Accordingly, myofibroblast differentiation of endometrial MSCs potentially contributes to fibrogenesis in endometriosis. Actually, MSCs derived from various tissues, such as lung, kidney and bone marrow, have been reported to promote the development of fibrotic diseases by differentiating into myofibroblasts [5–8]. Dense fibrosis in and surrounding the endometriotic lesions, a prominent histological feature of endometriosis, may lead to scarring, chronic pain, and altered tissue function [9]. Studies aimed at clarifying the underlying mechanisms of myofibroblast differentiation of endometrial MSCs are expected to provide a novel strategy for treatment of endometriosis.

MSCs, which are highly sensitive to the microenvironment, differentiate into myofibroblasts driven by appropriate local factors [10]. It is hypothesized that ectopic endometrial MSCs exist in the peritoneal cavity exposed to peritoneal fluid. Numerous soluble factors in endometriotic peritoneal fluid are abnormally expressed [11]. A cytokine array analysis revealed that 74 cytokines increased and four cytokines decreased with a threefold change in women with endometriosis. The increased multiple of activin A, up to 88.74 times, was the highest [12]. Activin A, originally recognized as an inducer of follicle-stimulating hormone release from the pituitary, has been found to play roles in a wide spectrum of physiologic and pathogenic events, such as cell proliferation, differentiation, apoptosis and metabolism dependent on the cell context [13, 14]. Recently, Activin A has also been reported to mediate inflammation, immunity, wound repair, and fibrosis [15]. We therefore speculate that Activin A may play a role in myofibroblast differentiation of endometrial MSCs. In this study, we investigated whether Activin A stimulated endometrial MSCs to differentiate into myofibroblasts and clarified the underlying mechanisms. We also employed the inhibitors of Activin A to explore the possibility of suppressing the development of fibrosis in endometriosis by blocking Activin A pathway.

## Materials and methods

### Patients and specimens

Thirty-two patients (mean age 34.4 years; range 23–49) with laparoscopically and histopathologically confirmed ovarian endometriosis and twenty control patients (mean age 35.0 years; range 24–47) surgically treated for benign gynecological conditions such as uterine leiomyoma and benign ovarian cyst, having no evidence of endometriosis at laparoscopy, were included in this study. All of the women had regular menstrual cycles and none of them had received hormonal treatment for at least 3 months prior to the surgery. Peritoneal fluid was aspirated from the cul-de-sac (Douglas) immediately after the establishment of the pneumoperitoneum and before any laparoscopic manipulation. Blood-contaminated peritoneal fluid was excluded. The peritoneal fluid were immediately cleared of cells and cell debris by centrifugation at 2000 rpm for 10 min at 4 °C, filtered through a 0.22 μm-pore size membrane and stored at − 80 °C. Eutopic endometrial biopsies were collected using endometrial suction catheters. Cyst walls of ovarian endometrioma were collected and ectopic endometrial tissues were carefully stripped from the lining inner cyst wall. Informed consent was obtained from all human subjects and the study was approved by the Institutional Review Board of Jiangsu Province Hospital on Integration of Chinese and Western Medicine (No. 2015LW032).

### Enzyme-linked immunosorbent assay (ELISA)

The concentration of Activin A in undiluted individual peritoneal fluid was measured in triplicate using a commercial specific ELISA kit (R&D Systems) according to the detailed protocol provided by the manufacturer.

### Isolation and identification of endometrial MSCs

Endometrial stromal cells were isolated as previously described [16]. The cells were incubated in ACK lysing buffer (Thermo Fisher) and then centrifuged to remove erythrocytes. Cells at passage 1 were harvested to extract endometrial MSCs by magnetic bead selection as described by Ulrich et al. [17]. Briefly, cell suspensions were labeled with the PE-conjugated SUSD2 (W5C5) antibody (Biolegend) in autoMACS running buffer (Miltenyi Biotec) at 4 °C for 15 min. After twice washing, the cells were incubated with anti-PE microbeads at 4 °C for 15 min. After washing, the cells were applied onto the column in a magnetic field and SUSD2+ cells were collected as endometrial MSCs. The isolated SUSD2+ MSCs accounted for 7.7% ± 3.9% of endometrial stromal cells. Cultured endometrial MSCs at passage 2 to passage 5 were used for the following experiments. These endometrial MSCs, with cloning efficiency 0.83–2.92%, were positive for CD44, CD73, CD90, CD105, and negative for CD34, CD45 by flow cytometric analysis (Additional file 1: Figure S1). The multipotency of endometrial MSCs was confirmed by their ability to differentiate into osteocytes and adipocytes using respective induction media (Additional file 2: Figure S2).

### Cell treatment

Cultured eutopic endometrial MSCs from patients without endometriosis were treated with 25 ng/mL recombinant human Activin A (R&D System), 25 ng/mL recombinant human CTGF (Peprotech), 0.18 μg/mL αActivin (R&D System), 10 μM Stattic, 10 μM SB431542 (Selleck) or 0.18 μg/mL Follistatin (R&D System) for indicated time. The cells were collected for q-PCR, Western blot analysis, immunofluorescence staining or chromatin immunoprecipitation (ChIP).

### Western blot analysis

Western blot analysis was performed as previously described [18]. Specific antibodies against ALK4, α-SMA, collagen I, CTGF, fibronectin, p-Smad3, GAPDH, Smad2/3, p-Smad2, p-Akt, Akt, p-p38, p38, p-JNK, JNK, p-STAT3, STAT3 and Histone H3 were used as primary andibodies. Secondary antibodies and immobilon Western chemiluminescent HRP substrate (Millipore) were used to visualize immunoactive bands. Primary antibodies used in this study were listed in Additional file 3: Table S1.

### RNA extraction and quantitative real-time polymerase chain reaction (qRT-PCR)

Total RNA was extracted using Column Animal RNAout kit (Tiandz) according to the manufacturer’s instructions. PrimeScript RT reagent Kit with gDNA Eraser (Takara) was used for reverse transcription-PCR reaction. Gene expression was quantified by QuantiNova SYBR Green PCR Kit (Qiagen) using the ABI Prim 7300 Sequence Detection System (Applied Biosystems). The relative expression of mRNA was calculated by normalization to GAPDH relative to the control.

### Vector construction and transfection

Lentiviral vector packing CTGF-specific shRNA were constructed (GeneChem). The sequence for CTGF-specific shRNA was 5′- CCGGAAATCTCCAAGCCTATCAAGTCTCGAGACTTGATAGGCTTGGAGATTTTTTTTC-3′; 5′- AATTGAAAAAAAATCTCCAAGCCTATCAAGTCTCGAGACTTGATAGGCTTGGAGATTT-3’ [19]. Moreover, LV-NC were purchased from GeneChem. Lentivirus transfection was performed as previously described [18].

### Immunofluorescent staining

Immunofluorescence analysis was performed as described previously [6]. Rabbit anti-Smad2/3 was used as primary andibody. Alexa Fluor 488-conjugated goat anti-rabbit antibody (Invitrogen) was used as secondary antibody. The nuclei were staining with DAPI (Sigma-Aldrich). The primary antibody was replaced by isotype IgG in the negative control. The images were captured using a confocal fluorescence microscope (Olympus).

### ChIP

The binding of Smad2/3 to the promoter of CTGF was examined using a ChIP assay kit (Thermo Fisher) according to the manufacturer’s instruction. Briefly, the crosslinked cells were lysed and the nuclei were digested in the presence of 25 U/mL micrococcal nuclease at 37 °C for 15 min. Then the digested chromatin was incubated with 0.37 μg/mL anti-Smad2/3 or anti-IgG antibody overnight at 4 °C on a rocking platform. DNA immunoprecipitated with anti-Smad2/3 or anti-IgG antibody was subjected to qRT-PCR assay. CTGF promoter-specific primers were used to amplify the Smad2/3 binding region. The primers were as follows: sense, 5′- TGGTGCTGGAAATACTGCGC-3′, antisense, 5′- ACATTCCTCGCATTCCTCCC-3’ [20].

### Animal and treatment

A total of 42 female BALB/c mice (8 weeks old, 18–21 g in weight), including 28 model (recipient) mice and 14 donor mice were used in this study. Endometriosis was induced using a previously described method [21]. One week before the endometriosis-inducing procedure, the mice were subcutaneously injected with estradiol valerate (0.2 mg/mouse). Then the donor mice were killed and their uteri horns were removed and put into a dish containing sterile saline. After peeling off the serosa and myometrium, the endometrium-rich fragments were finely chopped. The processed fragments were consistently smaller than 1 mm^3^. Fragments suspended in sterile saline were intraperitoneally injected into the recipient mice. Endometrial tissue fragments obtained from one mouse were injected to two mice. One day after the endometriosis-inducing procedure, animals were randomly assigned to four experimental groups of seven animals each. The mice received a daily intraperitoneally injection of recombinant mouse Activin A (R&D systems, AFL338, 0.7 μg/mouse), αActivin A, a neutralizing antibody of Activin A, (R&D systems, MAB3381, 3 μg/mouse), Activin A (0.7 μg/mouse) + αActivin A (3 μg/mouse) and saline, respectively. The treatment periods were 4 weeks in duration. Then the mice were sacrificed and the endometriotic lesions were harvested. All procedures carried out on animals were approved by the Animal Care and Use Committee of Jiangsu Province Academy of Traditional Chinese Medicine under the animal protocol number SYXK (Su) 2016–0018.

### Histology and immunohistochemistry

Masson trichrome stain and immunohistochemistry were performed according to common protocols [6]. For Immunohistochemistry, the primary antibodies were employed as follows: rabbit anti-CTGF, rabbit anti-α-SMA, rabbit anti-collagen I and mouse anti-fibronectin. The secondary antibodies incubated were horseradish peroxidase-conjugated goat anti-rabbit/mouse immunoglobulin G (Boster). The primary antibody was replaced by isotype IgG in the negative control. All the slides stained in the same staining were analyzed for histological quantification using Image ProPlus (version 6.0, Media Cybernetics) as described previously [22].

### Statistical analysis

In vitro data represent at least three independent experiments using cells from a minimum of three separate isolations. Data that followed a normal distribution were presented as means ± SD and analyzed by One-way ANOVA with LSD post hoc test. Data that were not normally distributed were presented as boxplots, in which the bottom and top of the box represent the lower and upper quartiles, respectively, the band near the middle of the box represents the median, and the ends of the whiskers represent the smallest and the largest non-outlier observations, and subjected to Mann-Whitney U-test. All these tests were performed using the statistical package SPSS 17.0. A *p* value of less than 0.05 was considered statistically significant.

## Results

### Activin a elevated in endometriotic peritoneal fluid and ectopic endometrial MSCs expressed elevated level of activin receptor-like kinase (ALK4)

**Fig. 1:**
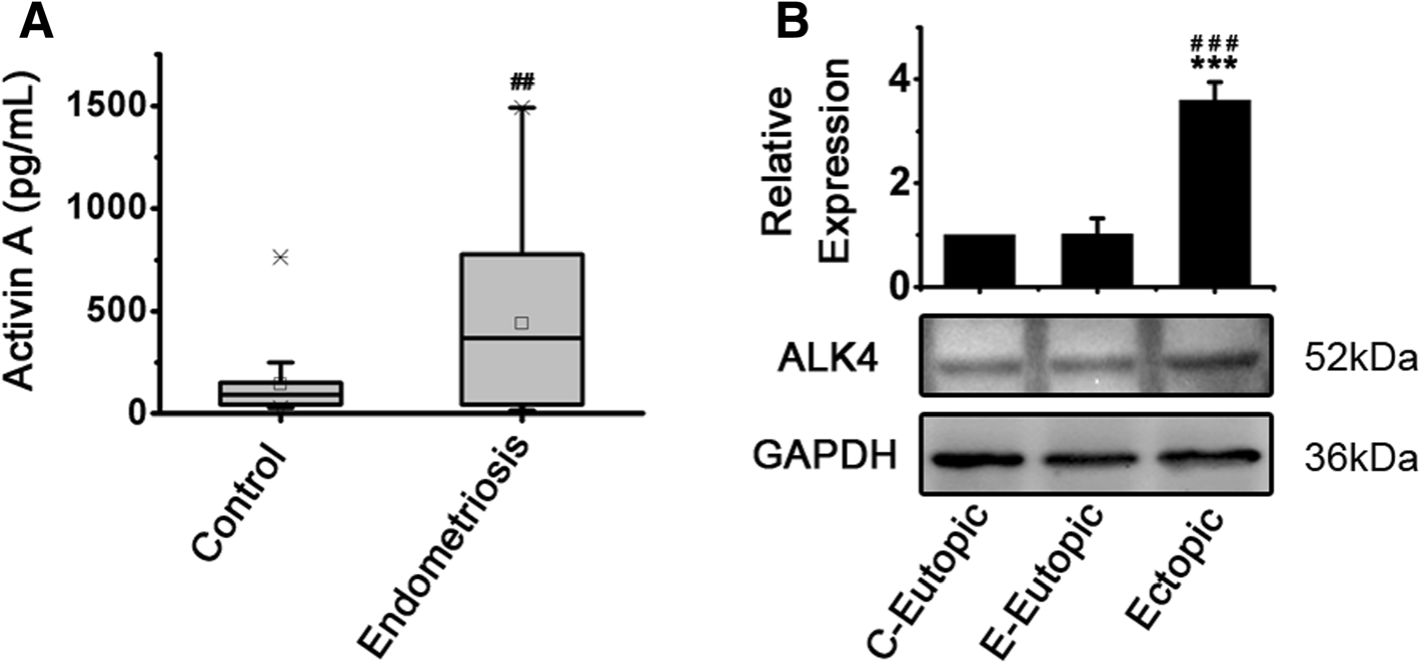
Activin A elevated in endometriotic peritoneal fluid and ectopic endometrial MSCs expressed elevated level of ALK4. **a** The concentrations of Activin A in the peritoneal fluid of women with (*n* = 32) and without (*n* = 20) endometriosis were measured by ELISA. ##*P* < 0.01 versus the control group. **b** The expression of ALK4 in paired eutopic and ectopic endometrial MSCs (passage 3) derived from patients with endometriosis (*n* = 3) and eutopic endometrial MSCs (passage 3) derived from patients without endometriosis (*n* = 3) was analyzed by Western blot. The expression levels of proteins were quantified by densitometry and normalized to the expression of GAPDH. The data were presented as means ± SD. ***P < 0.001 versus eutopic endometrial MSCs from control women (C-Eutopic). ###*P* < 0.001 versus eutopic endometrial MSCs from petients with endometriosis (E-Eutopic)

Hou et al. reported that Activin A increased to 88.74 times in peritoneal fluid of women with endometriosis when compared to women without based on a cytokine array analysis [12]. Because their samples for microarray analysis were from sample pools with three samples of each group, we expanded the sample size and determined the concentration of Activin A in individual peritoneal fluid using ELISA in this study. As shown in Fig. 1a, the concentration of Activin A significantly elevated in peritoneal fluid of women with endometriosis (*n* = 32 for endometriosis group and *n* = 20 for control group). We then compared the expression of ALK4 (also known as ActRIB), the specific receptor for Activin A, in cultured paired eutopic and ectopic endometrial MSCs derived from patients with endometriosis as well as eutopic endometrial MSCs derived from patients without endometriosis. The results showed that the expression of ALK4 obviously enhanced in ectopic endometrial MSCs compared with eutopic endometrial MSCs from women with or without endometriosis (Fig. 1b), implying the highly activated state of Activin A pathway in ectopic endometrial MSCs.

### Activin a induces myofibroblast differentiation of endometrial MSCs

**Fig. 2:**
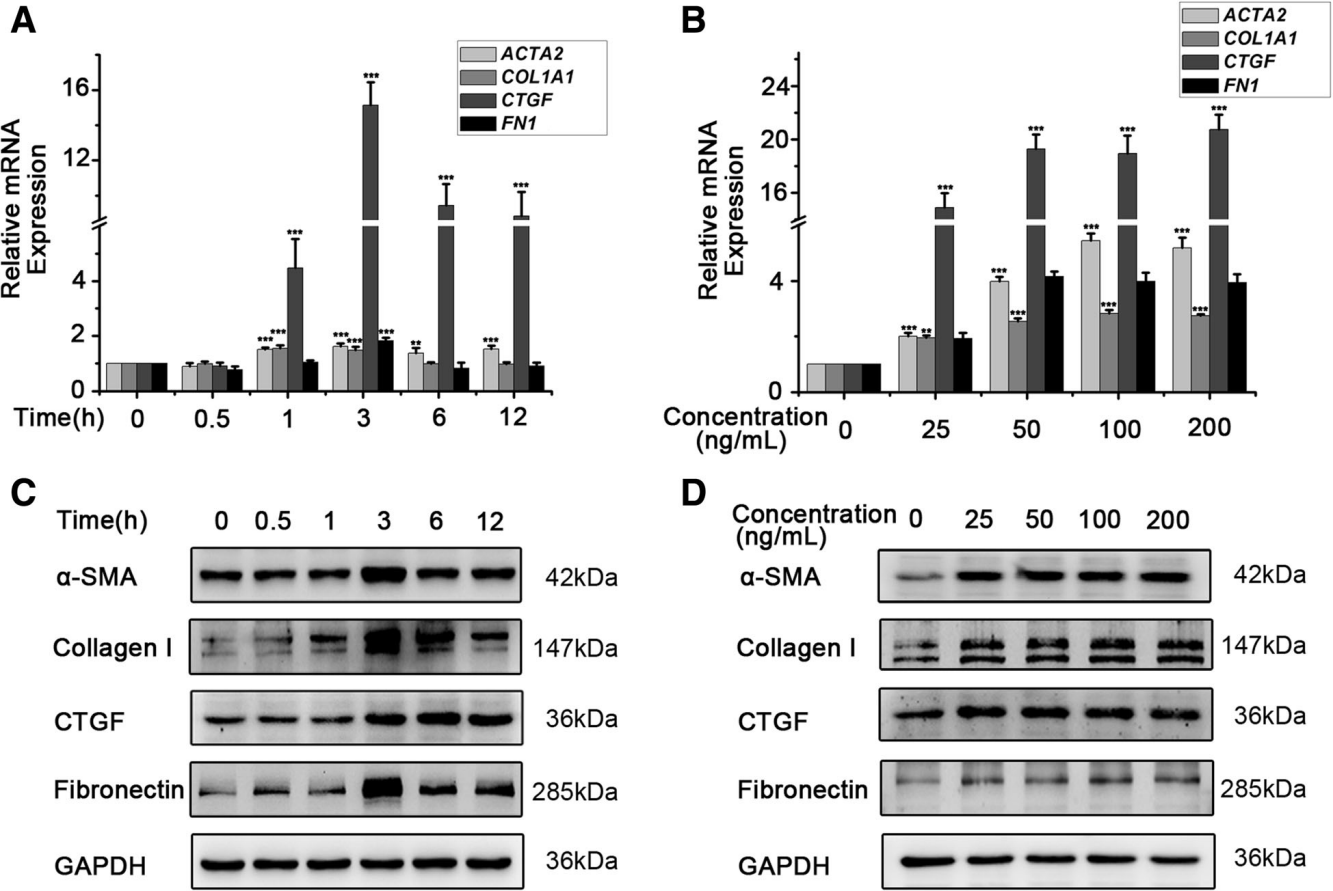
Activin A induces myofibroblast differentiation of endometrial MSCs. Eutopic endometrial MSCs derived from patients without endometriosis (*n* = 3) were treated with 25 ng/mL Activin A for indicated time (**a**) or with indicated concentration of Activin A for 3 h (**b**). mRNA expression levels of *ACTA2*, *COL1A1*, *CTGF* and *FN1* were analyzed by q-PCR. The data were presented as means ± SD. **P < 0.01 and ***P < 0.001 versus the cells treated without Activin A, respectively. Eutopic endometrial MSCs derived from patients without endometriosis (*n* = 3) were treated with 25 ng/mL Activin A for indicated time (**c**) or with indicated concentration of Activin A for 6 h (**d**). The protein expression of α-SMA, collagen I, CTGF and fibronectin were was analyzed by Western blot. The expression levels of proteins were quantified by densitometry and normalized to the expression of GAPDH. *P < 0.05 and ***P < 0.001 versus the cells treated without Activin A, respectively

To determine the effect of Activin A on myofibroblast differentiation of endometrial MSCs, we measured the expression of four fibrotic markers, including α-SMA, collagen I, fibronectin and CTGF, in cultured endometrial MSCs after treatment with recombinant human Activin A. As shown in Fig. 2b, d, Activin A (25 ng/mL-200 ng/mL) significantly promoted the expression of collagen I, α-SMA, CTGF and fibronectin at both mRNA and protein levels. 25 ng/mL Activin A induced the expression of collagen I, α-SMA, CTGF and fibronectin in a time-dependent manner (Fig. 2a, c). Interestingly, we found that the same-concentration Activin A up-regulated the expression of CTGF robustly while relatively weakly for collagen I, α-SMA and fibronectin at mRNA level (Fig. 2a, b), although collagen I and fibronectin proteins seemed to increase more than CTGF in endometrial MSCs in the time course (Fig. 2c).

### CTGF is essential for Activin A-induced expression of collagen I, α-SMA and fibronectin in endometrial MSCs

**Fig. 3:**
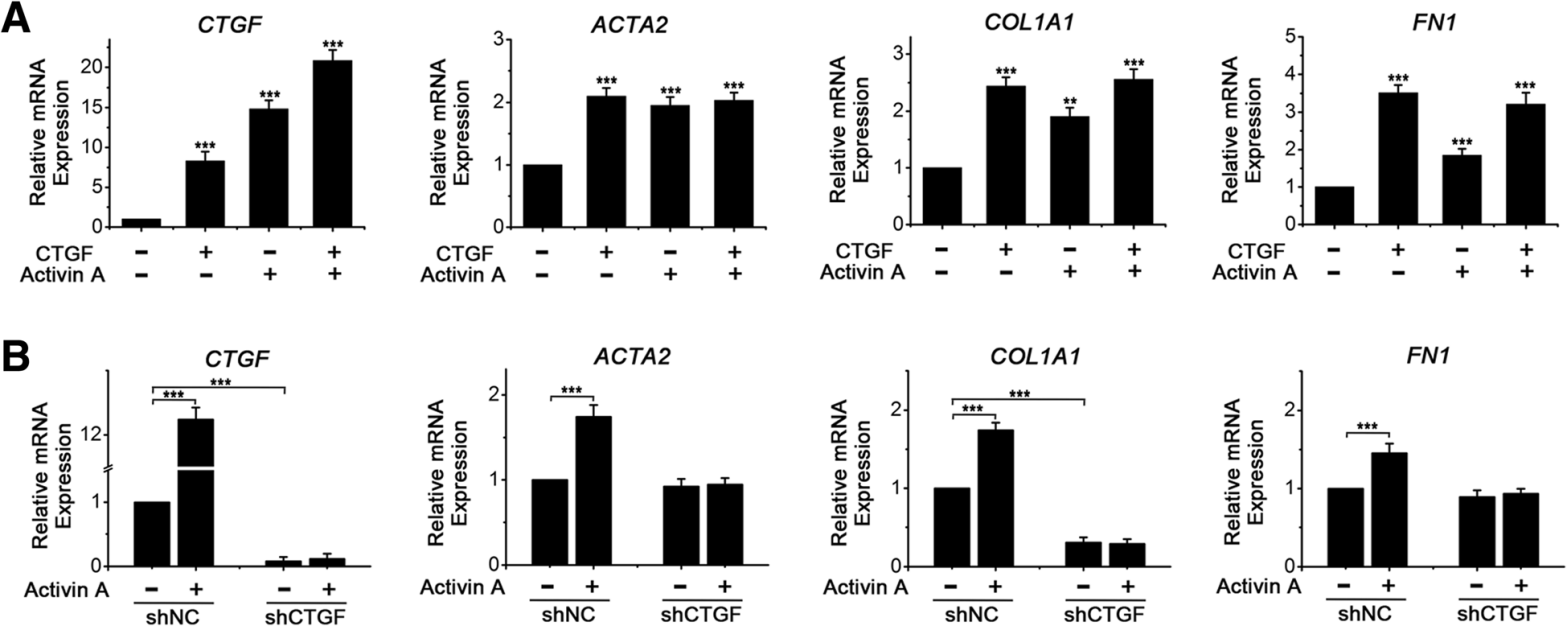
CTGF is essential for Activin A-induced expression of collagen I, α-SMA and fibronectin in endometrial MSCs. **a** Eutopic endometrial MSCs derived from patients without endometriosis (*n* = 3) were treated with recombinant human Activin A (25 ng/mL) and/or recombinant human CTGF (25 ng/mL) for 3 h. mRNA expression levels of *CTGF*, *ACTA2*, *COL1A1* and *FN1* were analyzed by q-PCR. The data were presented as means ± SD. **P < 0.01 and ***P < 0.001versus the cells treated neither Activin A nor CTGF, respectively. **b** Eutopic endometrial MSCs derived from patients without endometriosis (*n* = 3) were transfected with control shRNA (shNC) or shRNA targeting CTGF (shCTGF) and treated with 25 ng/mL recombinant human Activin A for 3 h. mRNA expression levels of *CTGF*, *ACTA2*, *COL1A1* and *FN1* were analyzed by q-PCR. The data were presented as means ± SD. *** represents *P* < 0.001

CTGF, a pro-fibrotic cytokine, has been recognized as an important player in the induction of matrix genes, such as collagen I, collagen III and fibronectin, to promote the development and maintenance of fibrosis [23, 24]. We then examined whether CTGF stimulated the expression of collagen I, α-SMA and fibronectin in endometrial MSCs and whether the expression of collagen I, α-SMA and fibronectin induced by Activin A in endometrial MSCs was dependent on CTGF. We added recombinant human CTGF (25 ng/mL) and/or recombinant human Activin A (25 ng/mL) into the cultures of endometrial MSCs and found that CTGF remarkably raised the expression of *ACTA2*, *COL1A1* and *FN1* even greater than Activin A (Fig. 3a). Intriguingly, CTGF also increased the expression of its own mRNA although the up-regulation effect is not as strong as Activin A (Fig. 3a).

Subsequently, lentiviral (LV) transfection of shCTGF was used to determine the role of CTGF in Activin A-induced expression of collagen I, α-SMA and fibronectin in endometrial MSCs. As shown in Fig. 3b, Activin A did not induce the expression of *ACTA2*, *COL1A1* or *FN1* in LV-shCTGF transfected cells although the expression significantly increased in cells transfected with negative control virus (LV-NC) after treatment with Activin A, implying that CTGF was essential for Activin A-induced expression of collagen I, α-SMA and fibronectin in endometrial MSCs.

### Activin A-induced CTGF expression in endometrial MSCs involves activation of Smad2/3

**Fig. 4:**
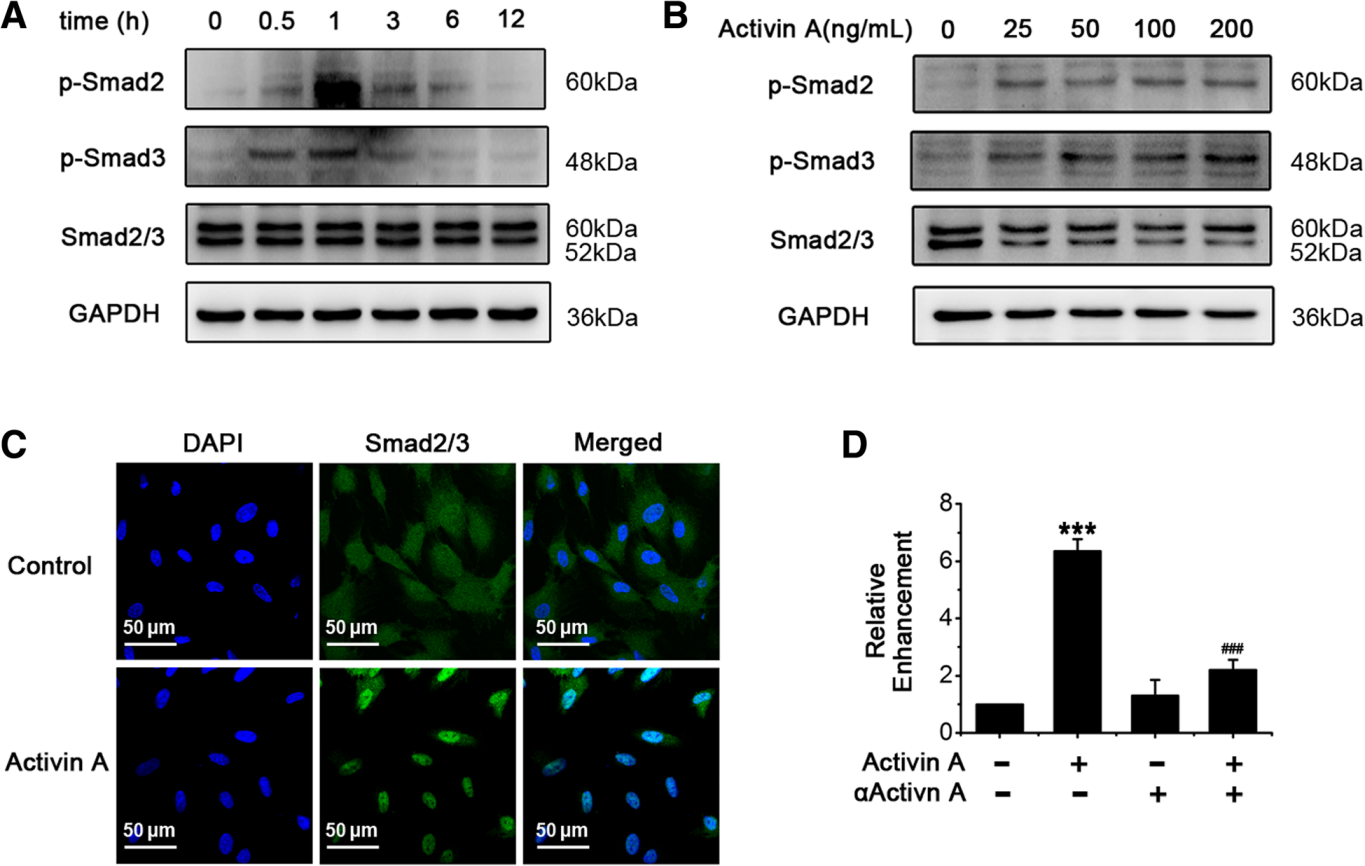
Activin A-induced CTGF expression in endometrial MSCs involves activation of Smad2/3. **a** Eutopic endometrial MSCs derived from patients without endometriosis (*n* = 3) were treated with 25 ng/mL Activin A for indicated time. The protein expression of p-Smad2, p-Smad3 and Smad2/3 was analyzed by Western blot. **b**, **c** Eutopic endometrial MSCs derived from patients without endometriosis (*n* = 3) were treated with 25 ng/mL recombinant human Activin A for 1 h. The protein expression of p-Smad2, p-Smad3 and Smad2/3 was analyzed by Western blot (**b**). The localization of Smad2/3 was examined by immunofluorescence analysis. The nuclei were stained with DAPI (**c**). **d** Eutopic endometrial MSCs derived from patients without endometriosis (*n* = 3) were treated with 0.18 μg/mL αActivin A for 1 h and then 25 ng/mL recombinant human Activin A for 1 h, ChIP was used to analyze the binding of Smad2/3 with CTGF promoter region. ***P < 0.001 versus the cells treated with neither Activin A nor αActivin A. The data were presented as means ± SD. ###*P* < 0.001 versus the cells treated with Activin A alone

When Activin A pathway is triggered, ALK4 phosphorylates Smad2/3 in cytoplasm and then Smad2/3 translocates into the nucleus to drive gene transcription [25]. In our study, Activin A induced phosphorylation of Smad2/3 (p-Smad2 and p-Smad3), both peaking at 1 h (Fig. 4a, b), in endometrial MSCs. Immunofluorescence showed that Activin A strongly induced nuclear translocalization of Smad2/3 (Fig. 4c). These results demonstrated that Smad signaling was activated in response to Activin A in endometrial MSCs. ChIP assays showed that Activin A promoted the binding of Smad2/3 to CTGF promoter substantially (Fig. 4d), consistent with the results that Activin A treatment up-regulated CTGF mRNA levels (Fig. 2a, b). The addition of αActivin A, a neutralizing antibody of Activin A, almost abolished the binding induced by Activin A (Fig. 4d). Our results indicated that Activin A promoted the transcription of CTGF through Smad2/3 in endometrial MSCs.

### STAT3 regulates Activin A-induced CTGF expression in endometrial MSCs through Smad2/3

**Fig. 5:**
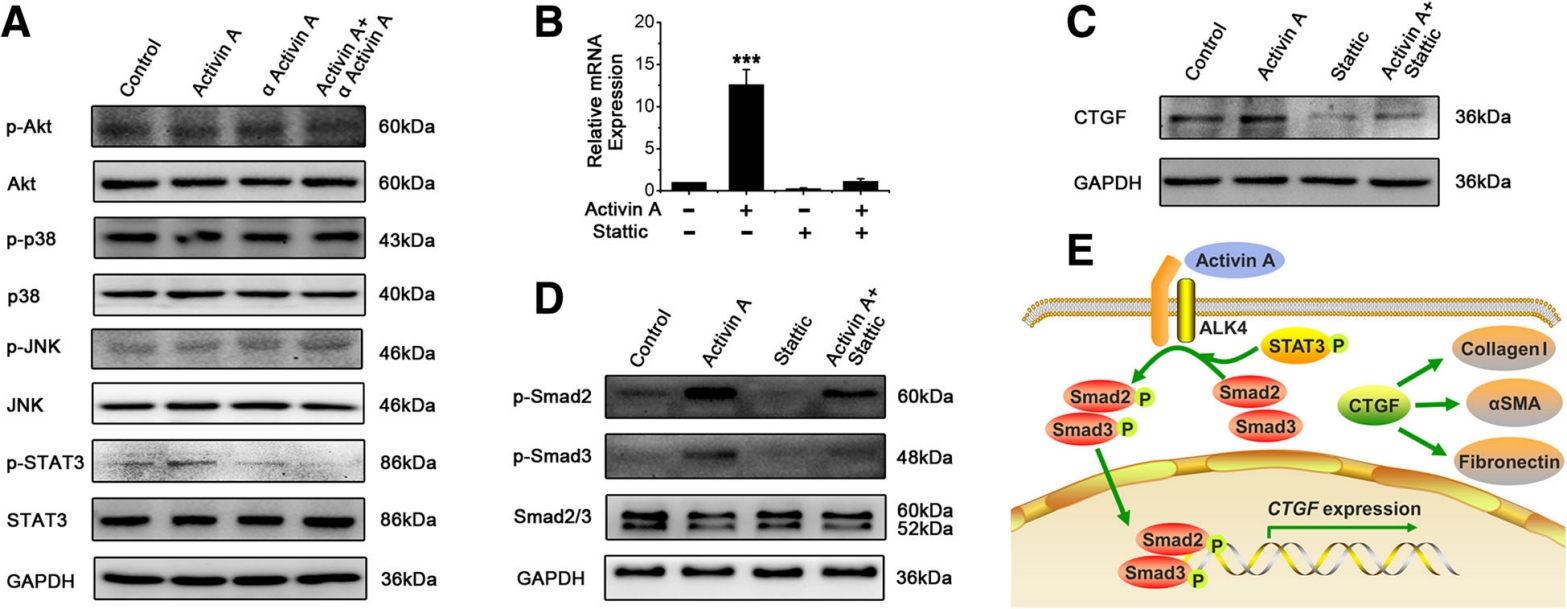
STAT3 regulates Activin A-induced CTGF expression in endometrial MSCs through Smad2/3. **a** Eutopic endometrial MSCs derived from patients without endometriosis (*n* = 3) were treated with 0.18 μg/mL αActivin A for 1 h and then 25 ng/mL recombinant human Activin A for 1 h. The protein expression of p-Akt, p-Akt, p-p38, p-38, p-JNK, JNK, p-STAT3 and STAT3 was analyzed by Western blot. **b**-**d** Eutopic endometrial MSCs derived from patients without endometriosis (*n* = 3) were treated with 10 μM Stattic for 1 h and then 25 ng/mL recombinant human Activin A for 3 h, the mRNA expression of CTGF was analyzed by q-PCR (**b**). After treatment with Activin A for 3 h, the protein expression of CTGF was analyzed by Western blot (**c**). The data were presented as means ± SD. ***P < 0.001 versus the cells treated with neither Activin A nor Stattic. The protein expression of p-Smad2, p-Smad3 and Smad2/3 was analyzed by Western blot (**d**). **e** Proposed model for Activin A-induced CTGF expression in endometrial MSCs

It has been reported that Akt, p-38, JNK and STAT3 signaling pathways also regulates the expression of CTGF [26–29]. In order to clarify the mechanism of Activin A induction of CTGF in human endometrial MSCs, we further examined the effects of Activin A on activation of Akt, p-38, JNK and STAT3. As shown in Fig. 5a, Activin A induced STAT3 phosphorylation while had no obvious effect on activation of Akt, p-38 or JNK pathways. αActivin A prevented phosphorylation of STAT3 caused by Activin A. Next, we used Sattic, a small-molecule inhibitor of STAT3, to evaluate the impact of STAT3 on the expression of CTGF stimulated by Activin A. The results revealed that Stattic significantly inhibit Activin A-induced up-regulation of CTGF expression (Fig. 5b, c). Given the fact that phosphorylated Smad2/3 could translocate into the nucleus and bind to the promoter of CTGF to facilitate the transcription (Fig. 4d), we then used Stattic to investigate whether STAT3 exerted an effect on activation of Smad2/3. We found that Stattic remarkably inhibited phosphorylation of Smad2 and Smad3 induced by Activin A in endometrial MSCs (Fig. 5d). These results indicated that Activin A-induced expression of CTGF via Smad pathway was STAT3-dependent.

### Inhibition of Activin a pathway impeded myofibroblast differentiation of endometrial MSCs and ameliorated fibrosis in endometriosis mice

**Fig. 6:**
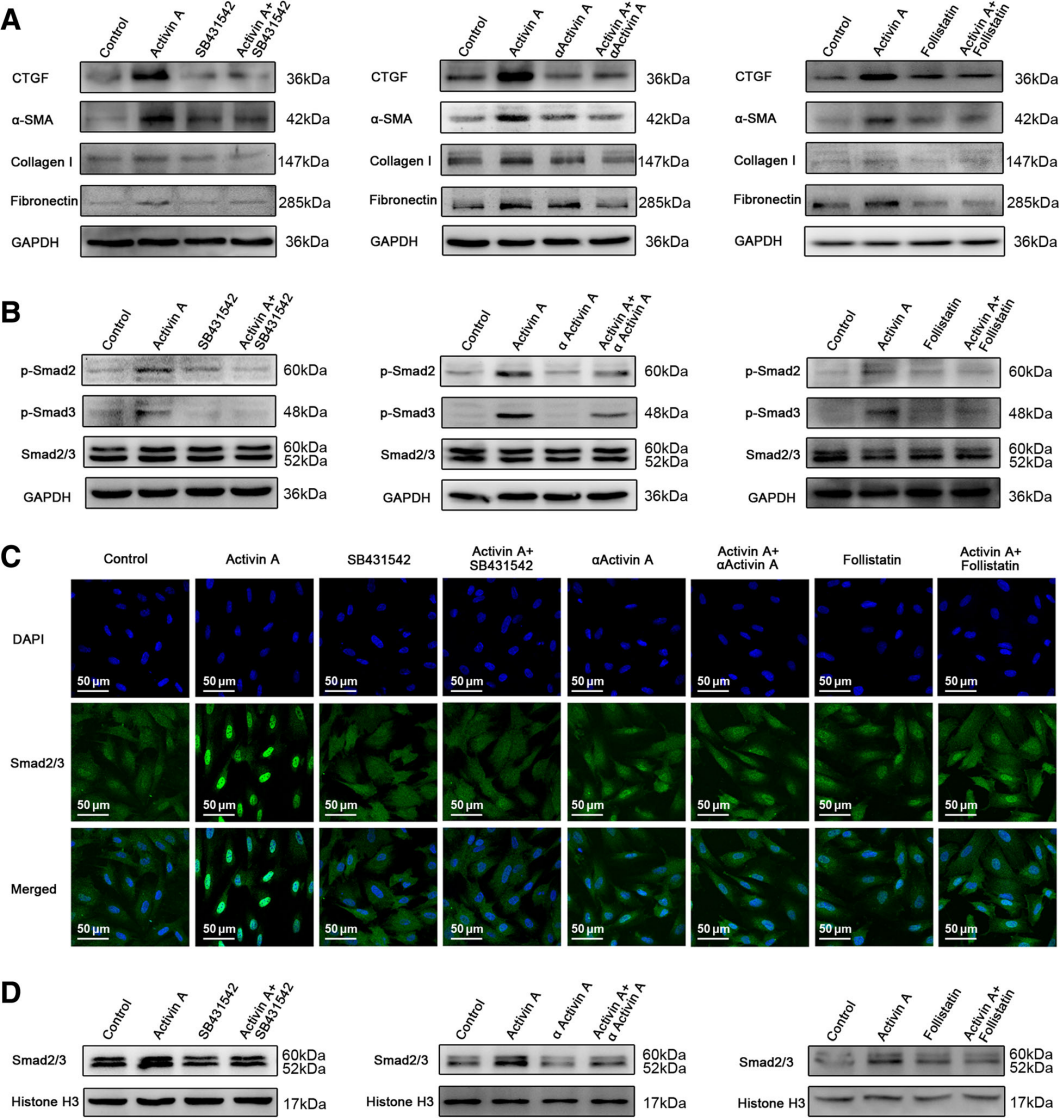
Inhibition of Activin A pathway impeded myofibroblast differentiation of endometrial MSCs. Eutopic endometrial MSCs derived from patients without endometriosis (*n* = 3) were treated with 10 μM SB431542, 0.18 μg/mL αActivin or 0.18 μg/mL Follistatin for 1 h and then treated with 25 ng/mL recombinant human Activin A. After treatment with Activin A for 6 h, the expression of CTGF, α-SMA, collagen I, fibronectin was analyzed by Western blot (**a**). After treatment with Activin A for 1 h, p-Smad2, p-Smad3 and Smad2/3was analyzed by Western blot (**b**). The localization of Smad2/3 was examined by immunofluorescence analysis. The nuclei were stained with DAPI (**c**). The expression of Smad2/3 in the nuclear extract was analyzed by Western blot (**d**)

To test whether blocking Activin A pathway could suppress myofibroblast differentiation of endometrial MSCs, we used the following three inhibitors of Activin A signaling. SB431542, inhibitor of ALK 4 and 5, was able to inhibit extracellular and intracellular Activin A signaling. αActivin A, neutralizing antibody of Activin A, and follistatin, endogenous antagonist of Activin A, were used to block extracellular Activin A signaling. Human recombinant Activin A was added to simulate the abnormally increased Activin A in endometriotic peritoneal fluid. As shown in Fig. 6a, all the three inhibitors prevented myofibroblast differentiation of endometrial MSCs by profoundly attenuating the expression of CTGF, α-SMA, collagen I and fibronectin. Moreover, the inhibitors could impair the phosphorylation (Fig. 6b) and nuclear translocalization (Fig. 6c, d) of Smad2/3. These results indicated that blocking Activin A signal notably impeded myofibroblast differentiation of endometrial MSCs along with the inactivation of Smad2/3.

**Fig. 7:**
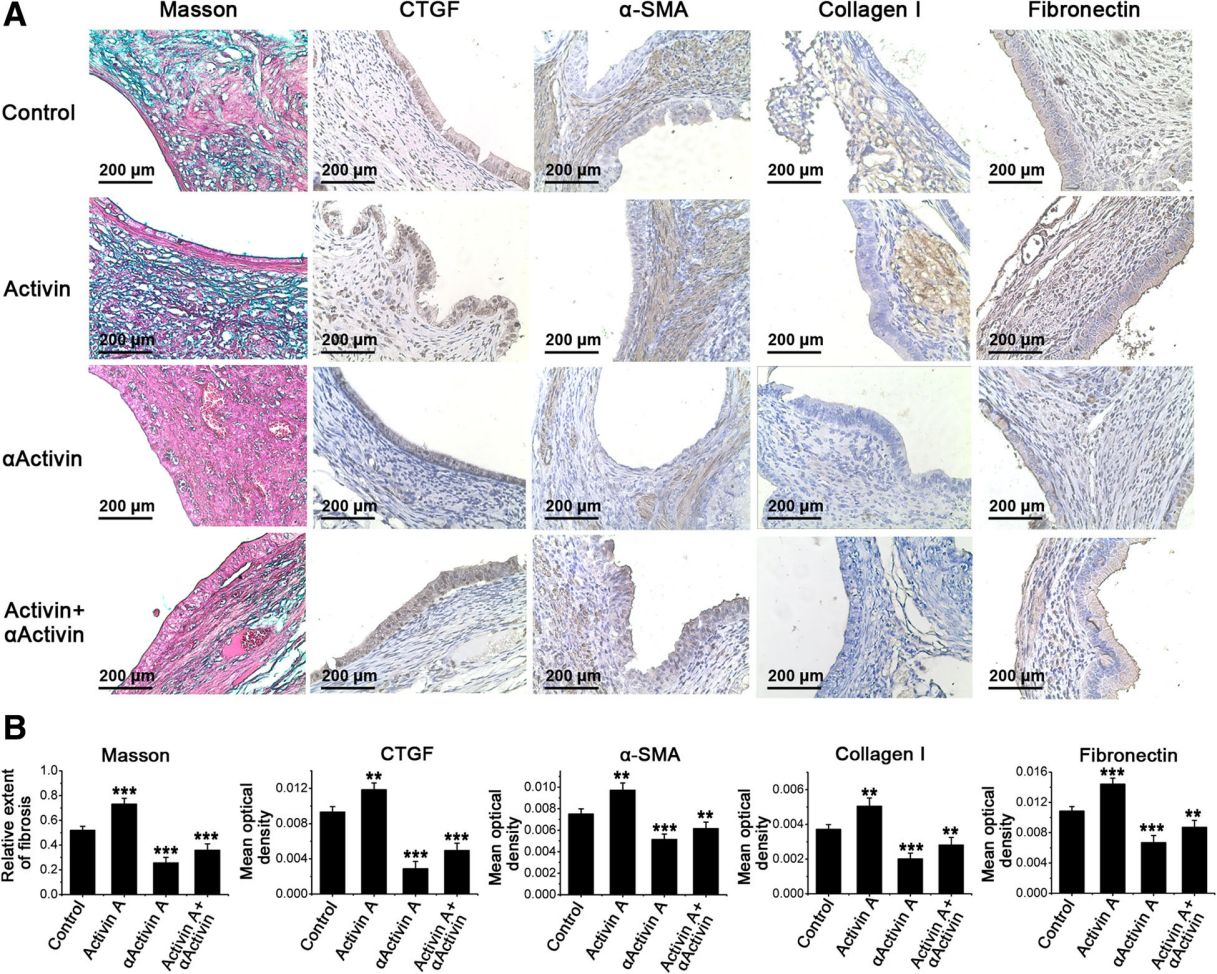
Inhibition of Activin A pathway ameliorated fibrosis in endometriosis mice. Endometriosis was induced in BALB/c mice. The mice were were randomly assigned to four experimental groups (*n* = 7) and received a daily intraperitoneally injection of recombinant mouse Activin A (0.7 μg/mouse), αActivin A, a neutralizing antibody of Activin A, (3 μg/mouse), Activin A (0.7 μg/mouse) + αActivin A (3 μg/mouse) and saline, respectively, for 4 weeks. Then the mice were sacrificed and the endometriotic lesions were harvested. **a** The collagen deposits and the expression of CTGF, α-SMA, collagen I and fibronectin in ectopic endometria were detected by Masson trichrome staining and immunohistochemistry, respectively. **b** Relative extent of fibrosis and mean optical density were determined by Image ProPlus. **P < 0.01 and ***P < 0.001 versus the control group, respectively

Having demonstrated the antifibrotic effect in vitro, we next explored whether blocking Activin A signal could modulate fibrosis in endometriosis in vivo. To address this question, we established a mouse model of endometriosis. As shown in Fig. 7, the fibrosis extent of ectopic endometria in Activin A group was obviously higher than that in control group, indicating that Activin A promoted the development of fibrosis in endometriosis in vivo. However, αActivin A significantly inhibited the excessive collagen deposition in ectopic lesions. The results of immunohistochemistry analyses showed that the expression levels of collagen I, α-SMA, fibronectin and CTGF in ectopic endometria were increased in Activin A group but decreased in αActivin A group compared with the control group. Meanwhile, αActivin A suppressed the up-regulation of collagen I, α-SMA, fibronectin and CTGF in ectopic endometria induced by Activin A.

## Discussion

In this study, we have demonstrated that Activin A induces myofibroblast differentiation of endometrial MSCs via STAT3-dependent Smad/CTGF pathway and that blocking Activin A signal impedes myofibroblast differentiation of endometrial MSCs in vitro and ameliorates fibrosis in endometriosis in a mouse model.

Patients with endometriosis possess altered peritoneal environment with numerous cytokines increased in peritoneal fluid [30]. A previous cytokine array analysis showed that the increase of Activin A was the most dramatically in endometriotic peritoneal fluid [12]. In the present study, we expanded the sample size to validate the result using ELISA. Our results demonstrated that the concentration of Activin A significantly elevated in peritoneal fluid of women with endometriosis though Florio et al. reported that peritoneal Activin A concentration was not significantly different between healthy women and patients with endometriosis [31]. The inconsistent results were perhaps caused by different types of endometriosis studied. Patients with ovarian endometriosis were included in our study while the specific type of endometriosis was not indicated in Florio et al.’s study. It has been reported that the concentration of Activin A in cystic fluid is higher than in peritoneal fluid in patients with ovarian endometriotic cysts [32]. Moreover, Activin βA subunit is strongly expressed in ovarian endometrioma [32, 33]. Ovarian endometriosis therefore may exhibit a higher concentration of Activin A in peritoneal fluid than other types of endometriosis. Activin A, a member of TGF-β superfamily, is involved in a variety of physiological and pathological processes. It is documented that Activin A stimulates the secretion of estradiol by improving the expression of aromatase P450 in eutopic endometrial stromal cells from patients with endometriosis [34]. Activin A increases invasion of endometrial stromal cells and epithelial cells into modeled peritoneum [35]. These findings suggest that Activin A is probably involved in the pathogenesis of endometriosis.

On the other hand, growing evidences have demonstrated that endometrial stem cells are crucial for the occurrence and development of endometriosis [2]. Ectopic endometrial MSCs are responsible for the remarkable proliferative and invasive properties of endometriosis [36]. The increased expression of ALK4 in ectopic endometrial MSCs implies the highly activated state of Activin A pathway. Several studies have shown that Activin A plays an important role in the differentiation of stem/progenitor cells derived from nonendometrial tissues, such as cerebrocortical neural progenitor cells, embryonic stem cells and adipose progenitors [37–41]. The effect of Activin A on differentiation of endometrial stem/progenitor cells remains unclear. In this study, by isolating endometrial MSCs followed by incubation with Activin A, we found that Activin A was able to drive the differentiation of endometrial MSCs into the myofibroblast lineage, evidenced by the expression of specific molecular markers of myofibroblasts. Myofibroblast, which when activated serves as the main producer of extracellular matrix, is the key cellular mediator of fibrosis [42]. In fibrotic diseases, such as lung fibrosis and kidney fibrosis, myofibroblasts are generated from myofibroblast transition of mesenchymal cells especially MSCs in addition to epithelial/endothelial-mesenchymal (EMT/EndMT) transition and circulating fibrocytes [6–8, 43]. It has been reported that EMT and fibroblast-myofibroblast transdifferentation (FMT) contribute to the presence of myofibroblasts and the development of fibrosis in endometriosis [44]. The findings in the present study suggest that the pro-fibrotic capacity of endometrial MSCs in the pathogenesis of endometriosis deserves much attention. Furthermore, a recent study reports the fibrogenic effect of endometrial MSCs through paracrine [45]. On the contrary, MSCs derived from various tissues including endometrium have been shown to play a role in alleviating fibrosis [46, 47]. It is noteworthy that these MSCs are allogeneic or autologous derived from another healthy tissue [46, 47]. However, tissue-resident MSCs in fibrotic diseases often promote the development of fibrosis [48]. We speculate that the pro-fibrotic microenvironment may change the biological behaviors of tissue-resident MSCs. This is consistent with our results that eutopic endometrial MSCs from patients without endometriosis differentiated into myofibroblasts in the presence of Activin A which was aberrantly increased in endometriotic peritoneal fluid.

Activin A has also been shown to accelerate the proliferation of renal interstitial fibroblasts and lung fibroblasts to promote fibrosis [49, 50]. In the present study, however, Activin A did not affect the proliferation of endometrial MSCs (data not shown), indicating the cell selectivity of Activin A. This is in accordance with the results of our recent study that peritoneal fluid from patients with endometriosis strongly induced myofibroblast differentiation of endometrial MSCs but exerted a minimal effect on their proliferation (In Press), suggesting that Activin A may act as a major bioactive factor in endometriotic peritoneal fluid. In addition to the fibrogenic effect on endometrial MSCs, Activin A also regulates the production of estradiol and inflammatory cytokines in endometrial stromal fibroblasts [34, 51], implying that Activin A promotes the development of endometriosis through multiple ways.

In endometrial MSCs, Activin A profoundly enhances the expression of CTGF. CTGF, also known as CCN family protein 2 (CCN2), has been identified as an important fibrotic marker in endometrial diseases including endometriosis and intrauterine adhesions [9, 52]. CTGF plays critical roles in the induction of ECM production. It has been reported that CTGF increases the expression of fibronectin by mesangial cells and the production of type I, type III and type IV collagen by mesangial cells and fibroblasts [23, 53]. In endometrial stromal fibroblasts derived from patients with adenomyosis, CTGF mediates TGF-β-induced collagen expression [54]. In the present study, CTGF remarkably raised the expression of *ACTA2*, *COL1A1* and *FN1* in endometrial MSCs. By shRNA-mediated CTGF knockdown, CTGF was shown to be required for Activin A-induced expression of *ACTA2*, *COL1A1* and *FN1* in endometrial MSCs.

Our results revealed that Activin A mediated the production of CTGF in endometrial MSCs through Smad signaling, which is consistent with previous reports on liver progenitor cells, hepatocytes and systemic sclerosis fibroblasts [25, 55, 56]. In addition to canonical Smad pathway, CTGF can be induced by other bioactive factors through Smad-independent pathways. PTEN increased CTGF in diabetes mellitus through Akt [28]. STAT3 pathway was required in Thrombin-induced CTGF expression in human lung fibroblasts [26]. Endothelin-1 induces CTGF synthesis in human lung fibroblasts via JNK/AP-1 pathway [27]. Epigallocatechin-3-gallate completely blocked TGFβ1-induced CTGF production by inhibiting phosphorylation of JNK and p38 MAPK in buccal fibroblasts [29]. In the current study, activation of STAT3 was required in Activin A-induced CTGF expression in endometrial MSCs. A previous study has shown that STAT3 is involved in modulating TGFβ-induced CTGF production in activated hepatic stellate cells. The process is independent of Smad2/3 phosphorylation but additionally modulated by JNK and PI3K pathways [57]. Although both Activin A and TGF-β belongs to TGF-β superfamily, they can activate different downstream signaling cascades even if in the same cell [58, 59]. In our study, the effect of STAT3 on Activin A-induced CTGF expression was still mediated by Smad2/3 in endometrial MSCs. Moreover, it was independent of PI3K, JNK and p-38 pathways. To the best of our knowledge, the present study demonstrates for the first time that STAT3 acts as a crucial Activin A downstream signaling mediator to regulate CTGF production. Taken together, we proposed a model in which STAT3 was activated in myofibroblast differentiation of endometrial MSCs with the stimulation of Activin A. Then, Smad2/3 was phosphorylated and shuttled into the nucleus where it binded to the promoter area of CTGF. The excessive CTGF further promoted the expression of fibrotic proteins collagen I, α-SMA and fibronectin (Fig. 5e).

Additionally, our results raise the possibility that inhibition of Activin A pathway may impede myofibroblast differentiation of endometrial MSCs and therefore ameliorat fibrosis in endometriosis. As expected, all the three inhibitors of Activin A signaling prevented myofibroblast differentiation of endometrial MSCs. In the mouse model of endometriosis, αActivin A, neutralizing antibody of Activin A, significantly inhibited the excessive collagen deposition and the expression levels of collagen I, α-SMA, fibronectin and CTGF in ectopic lesions. In addition, by classifying endometrial tissues into epithelial and stromal components according to cell morphology, we found that the expression of CTGF in ectopic endometrial stromal component decreased more obviously than that in epithelial component after treatment with αActivin A, suggesting that ectopic endometrial stromal component, in which endometrial SUSD2+ MSCs exist, are more responsive to Activin A-induced CTGF production. Meanwhile, we have to note the possibility that Activin A also induced CTGF expression in cells other than MSCs in ectopic lesions cannot be ruled out since we failed to track the ectopic endometrial MSCs.

## Conclusions

In summary, our findings reveal that Activin A induces myofibroblast differentiation of endometrial MSCs via STAT3-dependent Smad/CTGF pathway and that blocking Activin A signal ameliorates fibrosis in endometriosis. Thus, our data may supplement the stem cell theory of endometriosis and provide the experimental basis to treat endometriosis-associated fibrosis by manipulating Activin A signaling.

## Additional files

Additional file 1: **Figure S1.** Endometial MSCs possess MSC phenotypic characteristics. Paired eutopic (E-Eutopic) and ectopic endometrial MSCs (passage 5) derived from patients with endometriosis and eutopic (C-Eutopic) endometrial MSCs (passage 5) derived from patients without endometriosis were identified for the expression of positive MSC markers CD44, CD73, CD90 and CD105, and negative MSC markers CD34 and CD45. Black lines, cells stained with a matched isotype control; Red lines, cells stained with the indicated antibodies. (TIF 9576 kb)

Additional file 2: **Figure S2.** Endometial MSCs possess MSC multipotency. Paired eutopic (E-Eutopic) and ectopic endometrial MSCs (passage 5) derived from patients with endometriosis and eutopic (C-Eutopic) endometrial MSCs (passage 5) derived from patients without endometriosis were cultured in osteogenic differentiation media and adipogenic differentiation media, respectively. The osteogenic differentiation was detected with alizarin red, and the adipogenic differentiation was detected with Oil Red O. (TIF 6845 kb)

Additional file 3: **Table S1.** Primary antibodies used in this study (DOCX 17 kb)

## Abbreviations

ALK: Activin receptor-like kinase
ChIP: Chromatin Immunoprecipitation
CTGF: Connective tissue growth factor
ELISA: Enzyme-linked immunosorbent assay
GAPDH: Glyceraldehyde-3-phosphate dehydrogenase
JNK: c-Jun N-terminal kinase
MSCs: Endometrial mesenchymal stem cells
STAT3: Signal transducer and activator of transcription 3
SUSD2: Sushi Domain containing-2
TGFβ1: Transforming growth factor-β
α-SMA: α-smooth muscle actin
