# Supplementary figures and images for: Activin a promotes myofibroblast differentiation of endometrial mesenchymal stem cells via STAT3-dependent Smad/CTGF pathway
Source: Cell Commun Signal. 2019 May 17;17:45. doi: 10.1186/s12964-019-0361-3 (PMC6525394; doi:10.1186/s12964-019-0361-3)

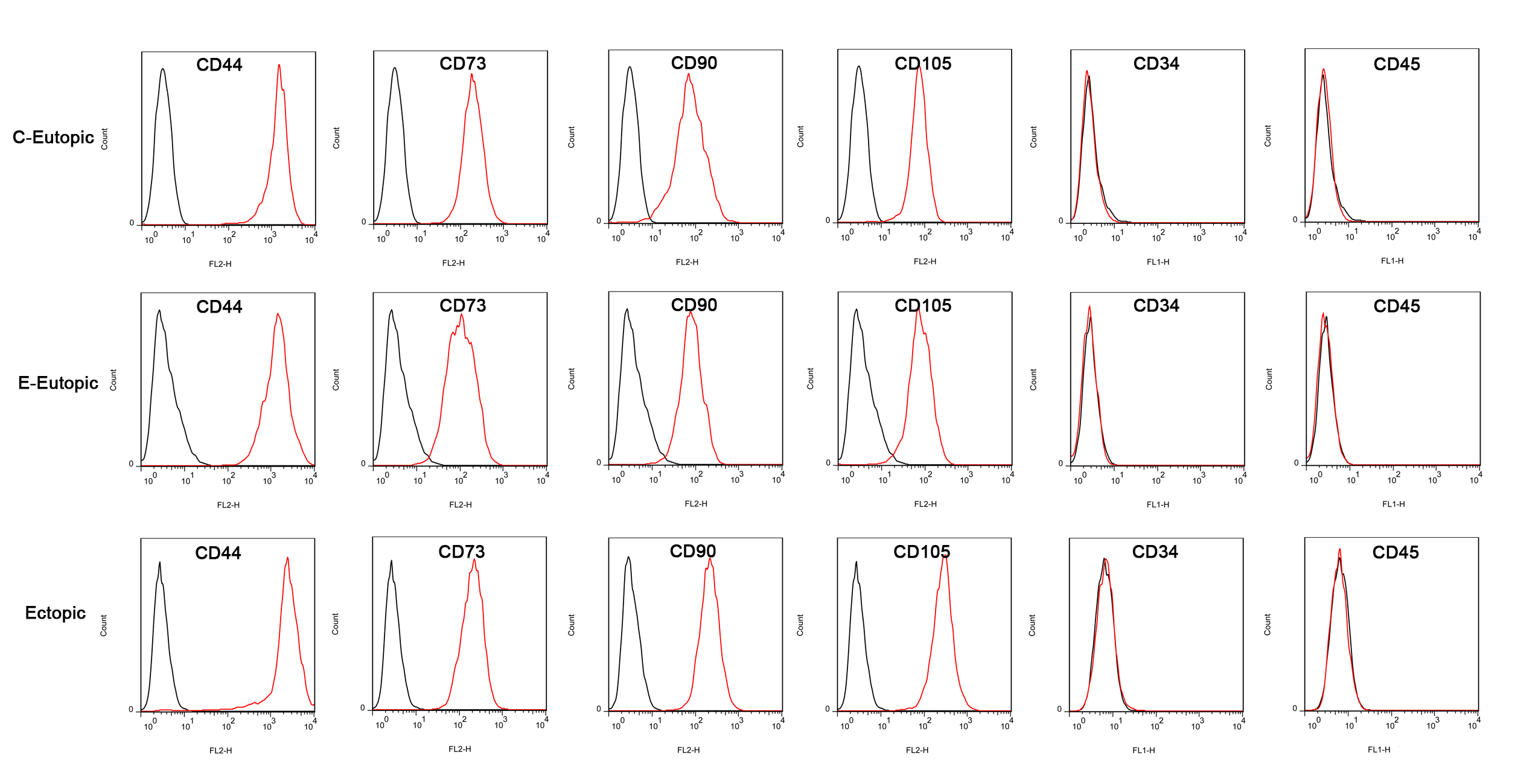

Supplement: Supplementary file 1 — Figure S1. Endometial MSCs possess MSC phenotypic characteristics. Paired eutopic (E-Eutopic) and ectopic endometrial MSCs (passage 5) derived from patients with endometriosis and eutopic (C-Eutopic) endometrial MSCs (passage 5) derived from patients without endometriosis were identified for the expression of positive MSC markers CD44, CD73, CD90 and CD105, and negative MSC markers CD34 and CD45. Black lines, cells stained with a matched isotype control; Red lines, cells stained with the indicated antibodies. (TIF 9576 kb) [file 12964_2019_361_MOESM1_ESM.tif]

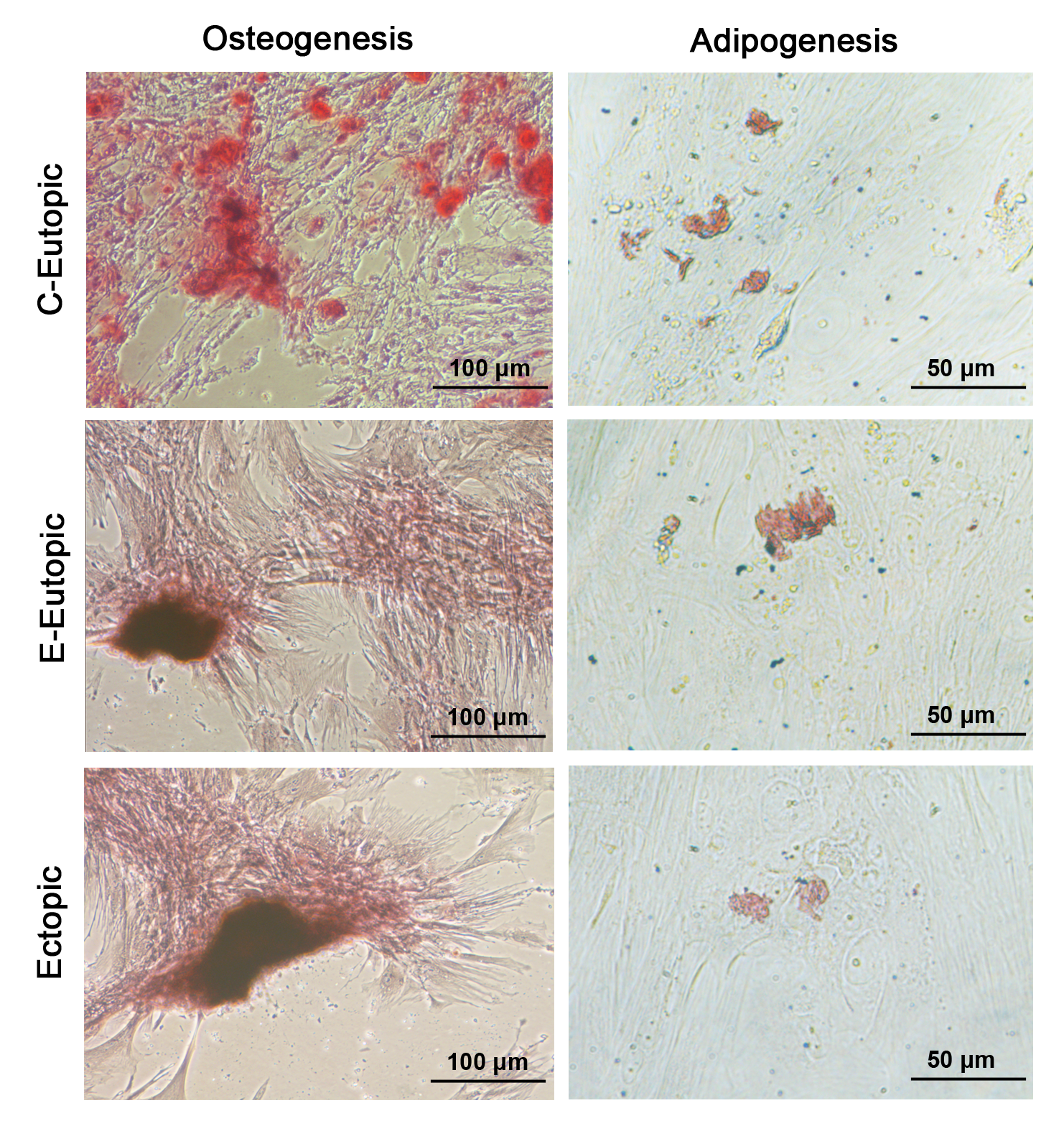

Supplement: Supplementary file 2 — Figure S2. Endometial MSCs possess MSC multipotency. Paired eutopic (E-Eutopic) and ectopic endometrial MSCs (passage 5) derived from patients with endometriosis and eutopic (C-Eutopic) endometrial MSCs (passage 5) derived from patients without endometriosis were cultured in osteogenic differentiation media and adipogenic differentiation media, respectively. The osteogenic differentiation was detected with alizarin red, and the adipogenic differentiation was detected with Oil Red O. (TIF 6845 kb) [file 12964_2019_361_MOESM2_ESM.tif]
