# Supplementary material for: Activin a promotes myofibroblast differentiation of endometrial mesenchymal stem cells via STAT3-dependent Smad/CTGF pathway
Source: Cell Commun Signal. 2019 May 17;17:45. doi: 10.1186/s12964-019-0361-3 (PMC6525394; doi:10.1186/s12964-019-0361-3)
Supplement: Supplementary file 3 — Table S1. Primary antibodies used in this study (DOCX 17 kb) [file 12964_2019_361_MOESM3_ESM.docx]

| Antibody | Manufacturers | source | Catalog No. | Clone No. | Dilution |
| --- | --- | --- | --- | --- | --- |
| ALK4 | R&D System | Mouse | MAB222 | 126822 | 1:1000 for WB |
| α-SMA | Abcam | Rabbit | Ab5694 | ／ | 1:1000 for WB |
| α-SMA | Boster | Mouse | BM0002 | 1A4 | 1:500 for IHC |
| collagen I | Abcam | Rabbit | ab34710 | ／ | 1:1000 for WB; 1:800 for IHC |
| CTGF | Abcam | Rabbit | ab6992 | ／ | 1:1000 for WB; 1:1200 for IHC |
| fibronectin | Abcam | Mouse | ab6328 | IST-9 | 1:1000 for WB; 1:1000 for IHC |
| p-Smad3 | Abcam | Rabbit | Ab52903 | EP823Y | 1:1000 for WB |
| GAPDH | Abcam | Mouse | ab8245 | 6C5 | 1:5000 for WB |
| Smad2/3 | Cell Signalling Technology | Rabbit | 8685 | D7G7 | 1:1000 for WB; 1:800 for IF; 1:100 for ChIP |
| p-Smad2 | Cell Signalling Technology | Rabbit | 3108 | 138D4 | 1:1000 for WB |
| p-Akt | Cell Signalling Technology | Rabbit | 4060 | D9E | 1:1000 for WB |
| Akt | Cell Signalling Technology | Rabbit | 4684 | 11E7 | 1:1000 for WB |
| p-p38 | Cell Signalling Technology | Rabbit | 4511 | D3F9 | 1:1000 for WB |
| p38 | Cell Signalling Technology | Rabbit | 8690 | D13E1 | 1:1000 for WB |
| p-JNK | Cell Signalling Technology | Rabbit | 4668 | 81E11 | 1:1000 for WB |
| JNK | Cell Signalling Technology | Rabbit | 9252 | ／ | 1:1000 for WB |
| p-STAT3 | Cell Signalling Technology | Rabbit | 9145 | D3A7 | 1:1000 for WB |
| STAT3 | Cell Signalling Technology | Mouse | 9139 | 124H6 | 1:1000 for WB |
| Histone H3 | Cell Signalling Technology | Mouse | 3638 | 96C10 | 1:1000 for WB |

Supplementary Table 1 Primary antibodies used in this study
